# Effect of Topical Administration of Fractions and Isolated Molecules from Plant Extracts on Skin Wound Healing: A Systematic Review of Murine Experimental Models

**DOI:** 10.1155/2016/4916068

**Published:** 2016-10-17

**Authors:** Mariáurea Matias Sarandy, Fernanda Barbosa Lopes, Sérgio Luis Pinto da Matta, Marcus Vinicius Mello Pinto, Sirlene Souza Rodrigues Sartori, Rômulo Dias Novaes, Reggiani Vilela Golçalves

**Affiliations:** ^1^Department of General Biology, Federal University of Viçosa, 35570-000 Viçosa, MG, Brazil; ^2^Department of Animal Biology, Federal University of Viçosa, 35570-000 Viçosa, MG, Brazil; ^3^Instituto Celulare, 25730-735 Petropolis, RJ, Brazil; ^4^Institute of Biomedical Sciences, Department of Structural Biology, Federal University of Alfenas, 37130-000 Alfenas, MG, Brazil

## Abstract

*Background and Purpose.* Skin wound healing is a dynamic process driven by molecular events responsible for the morphofunctional repair of the injured tissue. In a systematic review, we analyzed the relevance of plant fractions and isolates on skin wound healing. By revising preclinical investigations with murine models, we investigated if the current evidence could support clinical trials.* Methods.* Studies were selected in the MEDLINE/PubMed and Scopus databases according to the PRISMA statement. All 32 identified studies were submitted to data extraction and the methodological bias was investigated according to ARRIVE strategy.* Results.* The studies demonstrated that plant fractions and isolates are able to modulate the inflammatory process during skin wound healing, being also effective in attenuating the oxidative tissue damage in the scar tissue and stimulating cell proliferation, neoangiogenesis, collagen synthesis, granulation tissue expansion, reepithelialization, and the wound closure rate. However, we identified serious methodological flaws in all studies, such as the high level of reporting bias and absence of standardized experimental designs, analytical methods, and outcome measures.* Conclusion.* Considering these limitations, the current evidence generated from flawed methodological animal studies makes it difficult to determine the relevance of herbal medicines to treat skin wounds and derails conducting clinical studies.

## 1. Introduction

The skin wound healing is a dynamic and complex process divided into three complementary stages: inflammatory, proliferative, and maturation. The inflammatory phase comprehends the intense leucocytes recruitment to the wound area, removal of cellular and extracellular matrix debris, and syntheses of regulatory molecules such as cytokines and chemokines [1, 2]. The proliferative phase progresses with an intense proliferation and migration of fibroblasts, endothelial cells, and keratinocytes as well as formation of the granulation tissue (rich in type III collagen) and progressive reepithelialization [1–3]. At the maturation phase, type III collagen is gradually replaced by type I collagen, which originates more thicker and resistant collagen fibers [2–4].

It has been demonstrated that flaws on the leukocyte recruitment and function can impair the healing process due to reductions in the synthesis of regulatory molecules that drives the extracellular matrix assembly [5–7] and neoangiogenesis [8]. In this way, the development of drugs and alternative treatments that favor the migration and cellular activity during the inflammatory and proliferation phases may enhance the skin wound repair.

Skin wounds represent a serious health problem worldwide frequently associated with high costs and inefficient treatments [9, 10]. The use of herbal drugs is opening a new perspective for the treatment of skin wounds, mainly in developing countries. Once herbal strategies represent a simple pharmacological option, 80% of the population uses herbal drugs in their health care [1]. Although several plant species are currently used in the popular medicine to treat skin wounds worldwide [11–14], the scientific evidence that supports this practice is scarce. Thus, determining the security and efficiency of herbal drugs is an urgent and challenging task, which is essential to develop new technologies and products potentially applied in wound care.

In general, the healing properties of plant products are related to specific secondary metabolites, especially tannins, saponins, flavonoids, and alkaloids [11, 47, 48]. Plant products present a broad spectrum of biological functions such as astringent, antimicrobial, antioxidant, and anti-inflammatory [49–54] functions, which has been systematically associated with the beneficial effects in stimulating the healing process [49, 52, 54]. Before extrapolation to the human condition, preclinical researches using animal models have been useful for testing the toxicological security and biological effects of plant fractions and isolated molecules with potential applicability in the treatment of skin wounds [11, 52].

Despite the increasing number of experimental trials in the last decade, few advances were observed in the treatment of skin wounds, especially in humans. Considering that studies using animal models are conceived to support clinical investigations, there is a clear limitation in translating the findings obtained from animal models of wound healing to the human context. Considering that herbal drugs are extensively used in the popular medicine, we still do not know where the gap is that hinders the implementation of experimental findings for the development of innovations and technologies potentially useful in the clinical management of skin wounds. Thus, we systematically revised preclinical studies with murine models that investigated the effects of plant fractions and isolated molecules on the treatment of skin wounds. Beyond determining the relevance of plant derivatives in the skin repair, we analyzed the methodological quality of all preclinical studies identified, especially considering that the quality of evidence generated from flawed methodological studies could compromise the generalizability of the findings and derail conducting clinical studies.

## 2. Materials and Methods

### 2.1. Search Strategy

Research papers that investigate the action of plant fractions and isolated molecules in murine models of skin wound healing, published until 09/04/2015 (15:05:23), were recovered and independently analyzed by three researchers (FBL, MMS, and RVG). The search strategy was constructed by four components: “animals (filter),” “injury (wounds),” “organ (skin),” and “plants extract (isolates and fractions).” The filters were developed from PubMed database according to the hierarchical distribution of Medical Subject Headings [MeSH Terms]. A standardized search filter for animal studies was applied in PubMed database [55]. The same search strategy was adapted and used to recover studies in the Scopus platform. The standard animal filter provided by Scopus was used. The complete search strategy is described in Table S1 in Supplementary Material available online at http://dx.doi.org/10.1155/2016/4916068. Language restrictions were applied to recover only articles in English, Spanish, and Portuguese.

### 2.2. Selection Strategy

**Figure 1:**
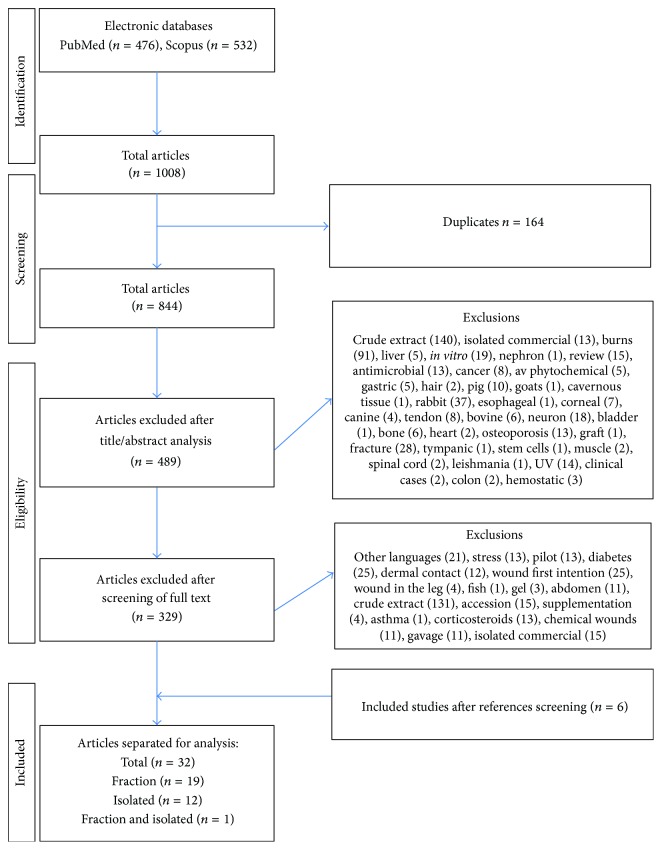
Flowchart of the strategy applied to recover preclinical studies according to the PRISMA statement.

An initial selection based on title and abstract [TIAB] was independently conducted by the researchers (FBL, MMS, and RDN). Duplicate studies were removed and only studies investigating the effect of fractions and isolated molecules from plant extracts in murine models of skin wound healing were considered. After the initial search, all relevant studies were recovered in full text and evaluated by eligibility criteria. Works containing unrefined extracts, commercial isolates,* in vitro* assays, humans, nontraumatic injuries, other animal models, first intention wounds, metabolic diseases associates, and secondary studies (i.e., letter to the editor, note, review, and editorial) were excluded (Figure 1).

### 2.3. Data Extraction

**Figure 2:**
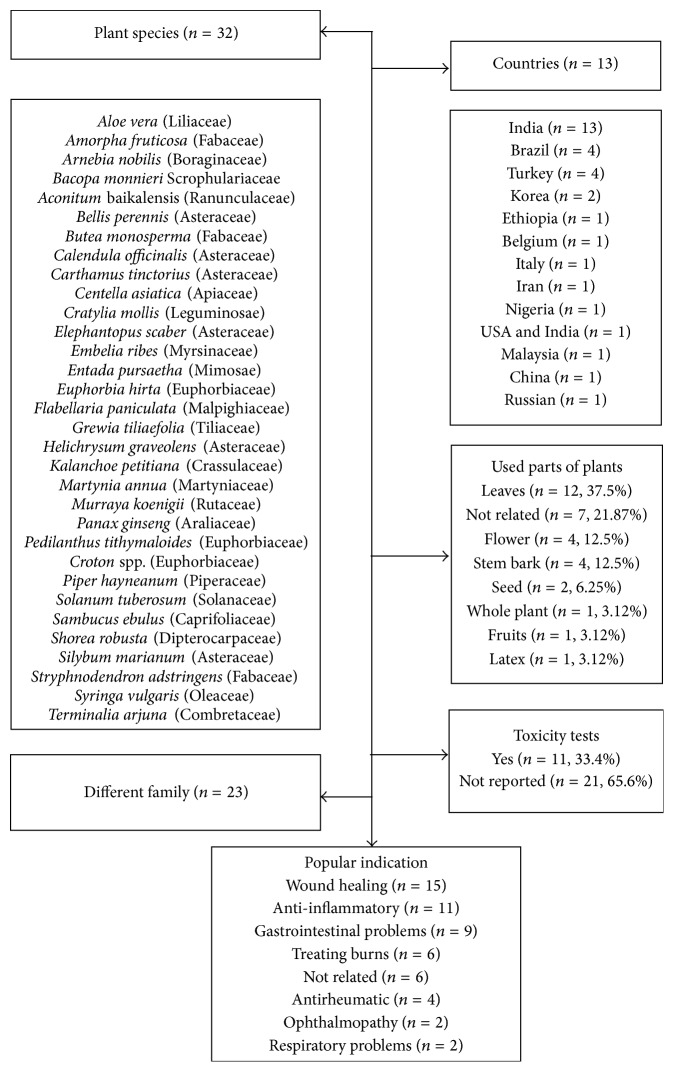
Summary of the studies describing the plants species, families, used parts of each species, toxicity tests, and popular indications.

Data were extracted and tabulated in a descriptive way (Tables 1(a), 1(b), 2(a), and 2(b)). The characteristics investigated were publication characteristics (author, title, publication year, and country); research methods (control group, randomization, experimental procedures, and blind evaluation of the results); experimental model (animal, number of animals, sex, age, weight, species, acclimation period, animal's housing, number of animals per cage and experimental groups, food supply, temperature, and light cycle); plants (plant's species, isolates, fractions, dose, toxicity test, exotic/native plant, popular name, utilized part of the plant, and popular indication); wounds description (wound area, measurement interval, and treatment duration) (Tables 1(a), 1(b), 2(a), and 2(b)). In a comprehensive approach, ethnobotanical/ethnopharmacological aspects were also investigated as follows: plant's species investigated (geographic distribution and existence or not of bioprospecting), popular indication, and reports of toxicity tests (Figure 2).

### 2.4. Analysis of Bias

The articles quality was analyzed by the criteria described on the ARRIVE platform (Animal Research: Reporting of* In Vivo* Experiments). These criteria are based on short descriptions that indicate essential characteristics of all studies with animal models, such as theoretical and methodological basis, research objective, refinement of the analytical methods, statistical design, sample calculations, and measure outcomes [15]. Recently there has been an increasing interest in the systematic reviews of research involving animals [16]. Considering the purpose of the systematic review on evaluating important aspects of the referenced publications, we built a table summarizing all the aspects investigated as well as their relevance, describing positive and negative characteristics of the recovered studies (Tables 2(a) and 2(b)).

## 3. Results

### 3.1. Included Studies

From the PubMed and Scopus database, 1008 articles were recovered. 164 duplicated studies and 489 with thematic inadequacy were excluded after reading the title and abstract. After recovery of 329 articles in full text, 303 studies were excluded for not meeting the eligibility criteria. Thus, 26 studies were included in the systematic review. The reference list of all included studies was carefully analyzed to ensure the identification of additional relevant studies. Thus, six studies were additionally identified and recovered, completing 32 works added to this review. From these studies, 19 studies utilized fractions, 12 studies utilized plant isolates, and 1 study used both fractions and isolates for the treatment of cutaneous wounds (Figure 1).

### 3.2. Qualitative Analysis

The analyzed studies were conducted in 13 different countries, especially India (40.62%, *n* = 13), followed by Brazil and Turkey (12.5%, *n* = 4 each). The most utilized animal models on the experiments were rats (75%, *n* = 24), followed by mice (12.5%, *n* = 4) and both (12.5%, *n* = 4). Considering the animal strain, 65.7% were Wistar rats, 17.14% were Sprague Dawley rats, 11.42% were Swiss mice, and 5.71% were Hairless mice. Half of the experimental models used male animals (*n* = 16), 15.62% (*n* = 5) used females, and 18.75% (*n* = 6) used both sex. 15.62% (*n* = 5) of all studies did not report this information. The animals' age ranged from 2 to 5 months for rats and from 8 to 12 weeks for mice; however 71.8% (*n* = 23) of the studies did not relate this information. The weight of rats ranged from 150 to 400 g and the mice weighted between 18 and 40 g, and only 2 studies (6.25%) did not report this data.

More than half of the studies did not describe the popular name of the plant species investigated (59.37%, *n* = 19). The first treatments utilized on the control group were as follows: 25% (*n* = 8) used ointment base (which did not have its formulation described), 15.6% (*n* = 5) used saline solution, 9.4% (*n* = 3) used nitrofurazone, and 6.2% (*n* = 2) utilized distilled water. Only 3.1% (*n* = 1) did not present the treatment for the control group. The other works utilized miconazole and nonionic cream; gentamicin; Matrigel solution; soft paraffin (85%), cetostearyl alcohol (5%), hard paraffin (5%), and wool fat (5%); framycetin ointment; PBS; sodium alginate; Vaseline; Tween 80; tragacanth; povidone iodine ointment; madecassol and ointment base; chlorocresol BP 0.1% mentioned only once, representing 40.6% of all included studies (*n* = 13). 62.5% (*n* = 20) of the plant species were native and 12.5% (*n* = 4) were exotic and 25% (*n* = 8) of the studies did not describe this characteristic.

Investigated wound area presented a large variation (5 mm^2^ to 600 mm^2^), and 9.37% (*n* = 3) of the studies did not describe this data. The calculations used to measure the wound area were described in only 59.37% (*n* = 19) of the studies. All the works described the interval in which the wound area was measured, and the most common interval was daily, 31.25% (*n* = 10), followed by measurements taken each 4 days, 28.12% (*n* = 9) (Tables 1(a) and 1(b)). From the 32 species of plants, 23 different families were reported, and the main ones are Asteraceae 18.75% (*n* = 6), Euphorbiaceae 9.37% (*n* = 3), Leguminosae 6.25% (*n* = 2), and Fabaceae 6.25% (*n* = 2), and the other families, Liliaceae, Boraginaceae, Scrophulariaceae, Ranunculaceae, Apiaceae, Myrsinaceae, Mimosae, Malpighiaceae, Tiliaceae, Crassulaceae, Martyniaceae, Rutaceas, Araliaceae, Piperaceae, Solanaceae, Caprifoliaceae, Dipterocarpaceae, Oleaceae, and Combretaceae, were mentioned once and represent 59.37% (*n* = 19) of the included studies. The most used plant structures were the leaves representing 37.5% (*n* = 12), followed by the flowers 12.5% (*n* = 4), bole bark 12.5% (*n* = 4), and seeds 6.25% (*n* = 2). The fruit, the whole plant, and the latex were mentioned once, representing 3.12% (*n* = 1) each. However, 21.87% (*n* = 7) of the studies did not mention this information. Considering the popular indication, healing effects were described in 46.87% (*n* = 15) of the studies, followed by anti-inflammatory effects 34.37% (*n* = 11), treatment of gastrointestinal diseases 28.12% (*n* = 9), burns 18.75% (*n* = 6), and antirheumatic 12.5% (*n* = 4) and ophthalmological diseases 6.25% (*n* = 2). 18.75% (*n* = 6) of the studies did not report the popular indication. Only 33.4% (*n* = 11) of the studies report toxicity tests (Figure 2).

### 3.3. Bias Analysis

Among the analyzed works, 78.12% presented a title coherent to the text, 90.6% presented abstracts containing the objectives, methods, main results, and conclusions, and 75% presented an introduction with sufficient scientific base. All studies described ethical approval and no work reported a blind controlled study. Most studies (87.5%) related to the therapeutic dose administered (90.62%) reported the route of administration and (96.87%) the treatment duration. The choice of administration route was not justified in any study. Most studies (96.87%) reported the investigated animal strain. The sex and weight were reported in 84.37% and 93.75% of the works, respectively, but only 31.25% provided information about the age of the animals. 59.37% of the studies provided information about the experimental conditions (temperature, humidity, light cycles, feed, and water). A statistical analysis was conducted by all studies, but only 68.75% of the studies specified the data analyzed. 84.37% of the studies reported the number of animals in each group. No study reported mortality or modifications on the experimental protocol by adverse events. A coherent interpretation of the results and direct relationship between objectives and hypothesis were described in 75% of all included studies (Table S2).

In general, the animals treated with isolates and fractions of plants presented an elevated closure rate of the wound, representing 72.72% of the studies [17, 18, 20, 23–27, 29, 30, 33, 35–46], increase in tissue reepithelialization (30.3%) [15, 18–20, 23, 27, 35, 38, 43], increase of the traction strength on the cicatricle tissue (75.75%) [15, 17–20, 22–25, 27, 31, 33–46], greater content and organization of the extracellular matrix on fast expansion of the granulation tissue (42.42%)  [17, 18, 23, 30, 32, 33, 36, 38–40, 42, 44–46], and stimulation of the activity of endogenous antioxidant enzymes (9.09%) [16, 21, 22] (Tables 2(a) and 2(b)).

## 4. Discussion

The use of plant based strategies is opening a new perspective for the treatment of skin wounds, mainly in developing countries, once it represents a simple, low cost, and affordable therapy [1, 7, 56–58]. There are several studies indicating beneficial effects of herbal medicines in all phases of the healing process. In fact, most of the studies included in this systematic review reported that plant fractions and isolates were able to improve the skin wound healing. Apparently, these medicines were especially favorable in controlling the cutaneous inflammatory and oxidative response and in stimulating the granulation tissue formation, collagen maturation, and reepithelialization.

In this review, we did not include studies testing crude plant extracts, since the chemical characterization of the extracts makes it difficult to determine the herbal components responsible for the effects reported. Even in case of including only studies with murine models, different animal strains were observed. This aspect makes the generalizability of the results difficult, since the biological variability directly influences the response to the treatments. In addition, among the 32 analyzed studies, there were large methodological variation and discrepancies in the measure outcomes. An evident example was the wide variation in wound area and time of wound closure. These considerations are important because they are directly associated with the tensile force experienced by the tissue, which profoundly affect the speed and quality of skin repair [59, 60]. Our findings show that 20% of the studies that utilized fractions neglected the analysis of wound closure, an essential piece of information to assess the ability of any intervention to stimulate the healing process. In addition, the interval between measurements of wound area and the used protocols for the calculations were variable, representing methodological flaws that compromise the study reproduction and generalizability of the findings [61, 62].

Considering that the reepithelization and organization of the granulation tissue are fundamental aspects to understand how chemical substances act to stimulate wound healing, only 60% of all studies analyzed the reepithelialization rate and 75% evaluated the molecular components of the extracellular matrix, especially collagen. These parameters indicate if the wound closure follows a normal process, in which the newly formed tissue gradually develops drastic structural changes to reconstitute the morphofunctional characteristics of the intact skin. Works which demonstrated the importance of these analyses assert that the type and quantity of collagen fibers deposited on the tissue can be used as a marker of tissue mechanical resistance [2, 3, 9, 58]. The connective and epithelial tissues form a support structure to promote the correct closure of the wound [57, 63], reducing the chances of opportunistic infections in the wounded area [38, 64]. During the formation of granulation tissue, there is predominance of sulfated molecules which attract water, facilitating the cellular migration, and also serve as a support structure for the first formed collagen (type III collagen) [65]. There are enough evidences that the synthesis and differentiation of cells and matrix components are crucial for a normal wound closure [60]. It is already known that the oxidative stress induces cell damage, lipid, protein, and nucleic acids oxidation [66, 67]. It is recognized that cutaneous trauma increases the tissue oxidative stress in the wounded area [66–69]. Although reactive species are able to activate cell signaling pathways and stimulate cell proliferation, differentiation, and neoangiogenesis, excessive production of these molecules inhibits the healing process, especially by inducing cell death and molecular damage in the extracellular matrix [23, 70, 71]. Thus, there is a notorious importance in analyzing the redox balance during skin repair. However, from all analyzed studies, only 15% investigated the oxidative status. This is a surprising finding, since the antioxidant effect is a pivotal mechanism indicated in several studies to support the applicability of plant extracts in the treatment of tissue damage, including skin wounds [66–69]. Another fundamental result on the cutaneous repair process is the restoration of the biomechanical properties, especially the tensile force of the newly formed tissue, which provides functional estimates on the quality of the healing process [37]. In this review, only 35% of all studies investigating plant fractions evaluated the traction resistance of the scar tissue, aspects investigated in 61.53% of the studies with plants isolates.

In our findings, we see that the majority of the studies used male animals, an aspect potentially associated with the hormonal stability, which is not observed in female animals due to the estrous cycle [72]. The use of rats as the experimental model was higher (75%), aspect potentially related to the large body area needed to perform experimental wounds (1 to 5). Thus, it is possible to construct a larger number of wounds and to use a smaller number of animals in each group. In addition, in rats it is possible to collect enough fragments in order to fully analyze the healing process. Another interesting piece of data was the age of the animals, which presented a large variation (rats, 2 to 8 weeks; mice, 5 to 12 weeks). However, 71.8% of the studies did not report this information, making it difficult to establish a temporal basis to determine the effectivity of the herbal treatments investigated. More than half of the studies (59.37%) did not describe the popular name of the plant species. The large number of works that did not describe important variables such as age of the animals and plant species represents a concerning number, once these characteristics are of great importance to ensure the study reproducibility and to allow the elaboration of broad reports with a critical review of the findings [15]. The orientation cited in the ARRIVE guideline describes the minimum information that all scientific publications using animals should include. This guide also brings items that help to understand the quality of the writing and potential methodological bias that compromise the quality of the evidence [15]. The work title should refer the readers to a brief summary of the article content, providing keywords and terms that could be researched in electronic databases [73]. Only 78.12% of the studies presented a coherent title, while 90.6% presented abstracts with clear information relative to the objectives, methods, main results, and conclusions. Furthermore, 75% presented introduction with enough scientific base, which can make it harder for the reader to understand the relevance of the study. Another observation made through ARRIVE guide refers to the health conditions of the animals during the experiment. Thus, aspects such as information about environmental conditions (temperature and humidity), mortality, feeding, randomization, and reactions indicative of systemic or local toxicity were neglected in most studies, demonstrating that the report bias is a serious limitation of these preclinical tests that compromise the reliability of the results and the quality of the evidence [74].

## 5. Conclusions

The current evidence indicates that fractions and isolated molecules from plant extracts stimulate the healing process in cutaneous wounds. Apparently, the main effects of these herbal medicines are associated with the stimulation of collagen synthesis, expansion of the granulation tissue, reepithelialization, modulation of the inflammatory response, and oxidative stress during tissue repair. Together, these effects promote increase of the speed of wound closure and the biomechanical resistance of newly formed tissue. However, the serious methodological flaws and report bias observed in most included studies make the current evidence fragile. Thus, the relevance of fractions and isolated molecules from plant extracts in the treatment of skin wound cannot be accurately determined. Considering these limitations, it seems impossible to use these evidences to construct a rational basis that supports clinical studies. Therefore, there is an urgent need to improve research reports in experimental studies with herbal medicines in murine models of skin wound healing. This task requires a collective effort of authors, journal editors, reviewers, and financial organisms, to ensure the reproducibility, reliability, and generalizability of the evidence, fundamental elements to determine to what extent herbal medicines are promising in the treatment of skin wounds.

## Supplementary Material

The full search strategy is presented in Table S1. Keywords were obtained in the MeSH (Medical Subject Headings) database, which is the U.S. National Library of Medicine (NLM) controlled vocabulary thesaurus used for indexing manuscripts in PubMed. In the additional databases used to recover relevant articles, the keywords were adapted acording the search algoritm adopted in the search plataforms.
Table S2 shows the results of bias analysis. All criteria investigated were based on ARRIVE guideline, which states the essential elements that should be reported in *In Vivo animal *experiments.

## Figures and Tables

**(a):** 

Reference	Country	Animal strain	Animal number	Sex	Age	Weight	Experimental groups	Animal number per group	Numbers of animals per box	Treatment control group	Plant species	Native/exotic	Used parts	Fractions	Dose	Popular indication	Wound area	Measurement interval	Wound area calculation	Treatment time
Bastos et al., 2011 [15]	Brazil	Wistar rats	12	♂	4 mo	300–320 g	4	3	1	Miconazole and nonionic cream	*Piper hayneanum* (Piperaceae)	?	L	[CHCl_3_-EtOAc 1 : 1 (haulm, A) and CHCl_3_-MeOH 1 : 1 (routs, B)	?	Anti-inflammatory, infectious skin diseases and healing, wounds, hematoma, and ecchymosis	6 mm^2^	Daily	?	15 days

Shukla et al., 1999 [16]	India	Sprague Dawley rats	?	♂	?	200–220 g	3	?	1	Saline solution	*Centella asiatica *(Apiaceae)	?	?	Asiaticoside	20 *μ*L of 0.2%	Healing activity	8 mm^2^	7/7 days	?	14 days

Muralidhar et al., 2011 [17]	India	Wistar rats	42	?	?	150–200 g	7	6	?	Ointment base	*Butea monosperma* (Fabaceae)	N	Sb	PETFR: petroleum ether fraction BENFR: benzene fraction, CHLFR: chloroform fraction, and ACEFR: acetone fraction	200 mg/kg-	Antitumor, antiulcer, antifungal, and antidiarrheal activities	500 mm^2^	4/4 days	?	16 days

Süntar et al., 2013 [18]	Turkey	Sprague Dawley rats	?	♂	?	160–180 g	?	6	?	Ointment base	*Helichrysum graveolens* (Asteraceae)	N	F	Hg-hexane; Hg-CH_2_Cl_2_; Hg-EtOAc, Hg-BuOH; Hg-R-H_2_O; Hg-Fr.B1; Hg-Fr.B2, Hg-Fr.B3, Hg-Fr.A, Hg-Fr.B; and Hg-Fr.C	0.5 g	Antimicrobial, antioxidant, anti-inflammatory, sedative, antidiabetic, and cytotoxic activities	5 mm^2^	Daily	Reduction in wounded area, using AutoCAD program	12 days

Mekonnen, et al., 2013 [19]	Ethiopia	Swiss mice/Wistar rats	24	?	8–10 wk/3–5 mo	30–40 g/180–200 g	4	6	1	Sodium carboxyl methyl cellulose xerogel and nitrofurazone	*Kalanchoe petitiana* (Crassulaceae)	N	L	Methanolic and chloroform fractions were 16%, 8.76%, 7.5%, and 5.6%, respectively	?	Wound healing, hemorrhoids, and antibacterial activities	312 mm^2^	Daily	% wound contraction = wound area on day 0 − wound area on day *n*/wound area on day 0 × 100	10 days

Pieters et al., 1995 [20]	Belgium	Wistar rats	?	♀	?	250–300 g	20	2	1	Not treated	*Croton *spp. (Euphorbiaceae)	E	Lx	Polyphenolic: PEG ointment, PEG 400 10%	0,5 mL/2x day	Wound healing	3 cm	Daily	?	18 days

Korkina et al., 2007 [21]	Italy	Wistar rats	40	♂	?	350–400 g	5	10	?	Saline solution	*Syringa vulgari*s (Oleaceae)	N	F	Two phenylpropanoid glycosides: verbascoside and teupolioside	100 *μ*L (0.2 mg/mL)	Wound healing, anti-inflammatory, antirheumatic, antipyretic, and antifungal activities	2.25 cm^2^	4/4 days	The recorded wounds were measured by planimetry using special computer program	8 days

Bigoniya et al., 2013 [22]	India	Wistar rats	30	♂	?	175 ± 10 g	5	6	?	Vehicle (not related)	*Euphorbia hirta* (Euphorbiaceae)	N	Wp	Flavonoid fraction (EHTF)	?	Antimicrobial, antifungal, antiviral, anti-inflammatory, antiarthritic, and antioxidant activities	500 mm^2^	4/4 days	?	20 days

Lodhi et al., 2011 [23]	India	Wistar rats	30	♂♀	?	150–200 g	5	6	1	Not treated	*Martynia annua* (Martyniaceae)	N	L	*M. annua* fraction: MAF-A, MAF-B, and MAF-C	?	?	500 mm^2^	2/2 days	% Wound contraction = healed area/total wound area × 100	20 days

Tabandeh et al., 2013 [24]	Iran	Wistar rats	60	♂	?	200 ± 50 g	4	15	?	Saline solution	*Silybum marianum *(Asteraceae)	E	?	Flavonoid silibinin (SB)	10% and 20% SB powder	Hepatoprotective and liver regenerating activities	1 cm	Daily	?	30 days

Sonmez et al., 2015 [25]	Turkey	Wistar rats	24	♂	?	180–260 g	3	8	?	Saline solution	*Solanum tuberosum *(Solanaceae)	?	?	Polysaccharide hemostat (APH)	3 mg of wheat meal in group 2 and 3 mg of APH in powder form	?	2 × 2 × 2 cm	3, 7, and 14 days	Percentage of contraction = [100 − (total wound area on the 14th day/total wound area on the 3rd day) × 100]	14 days

Karakaş et al., 2012 [26]	Turkey	Wistar rats	12	♂	?	200–250 g	2	12 and 8	?	Not treated	*Bellis perennis *(Asteraceae)	E	F	n-Butanol fraction	?	Activities in sore throat, headache, eczema, skin boils, and gastritis	4 mm^2^	1, 5, 10, and 30 days	Percentage of wound area = wound area in day/wound area in the first day × 100; percentage of wound healing = 100 − percentage of wound area	30 days

Choi et al., 2001 [27]	Korea	Hairless mice	10	♂	?	?	2	10	?	Vehicle (not related)	*Aloe vera *(Liliaceae)	?	?	Glycoprotein fraction named G1G1M1DI2	10 mg/g ointment Gentamicin 0.1%, every day	Wound healing, thermal injury healing, anti-inflammation, and immunomodulation activities	154 mm^2^	Daily	?	8 days

Parente et al., 2011 [28]	Brazil	Wistar rats	36	♀	60 days	160–190 g	2	18 and 6	1	Distilled water	*Calendula officinalis *(Asteraceae)	E	F	DCF: dichloromethane fraction at 1%; HCF: hexane fraction at 1%	?	Anti-inflammatory, first-degree burns, and skin rashes activities	1 cm	4, 7, and 14 days	?	14 days

Olugbuyiro et al., 2010 [29]	Nigeria	Wistar rats	16	♂	?	250–300 g	2	4	1	Gentamicin and saline solution	*Flabellaria paniculata *(Malpighiaceae)	N	L	Chloroform fraction and aqueous fraction	100 mg/mL	Activities in skin infections, wounds and sores, and dysentery	2 × 2 cm	7, 12, 14, and 18 days	?	18 days

Süntar et al., 2010 [30]	Turkey	Sprague-Dawley rats/Swiss mice	?	♂	?	160–180 g/20–25 g	9	6	?	Not treated	*Sambucus ebulus *(Caprifoliaceae)	N	L	Polyamide column fractions from the methanolic extract (Fr A, B, C, D, and E)	0,5 g	Hemorrhoids, rheumatic pain, treating burns, infectious wounds, edema, eczema, urticarial, and inflammations	5 mm^2^	2/2 days	Wound contraction was calculated as percentage of the reduction in wounded area	12 days

Kim et al., 2013 [31]	Korea	Hairless mice	10	♀	2 mo	?	2	5	?	Matrigel solution	*Panax ginseng *(Araliaceae)	?	L	Ginsenoside Rd	10 mL	Strengthening immune system and atherosclerosis activities	?	3/3 days	?	9 days

Chaudhari et al., 2006 [32]	India	Wistar rats	30	♂♀	?	180–250 g	5	6	?	Soft paraffin (85%), cetostearyl alcohol (5%), hard paraffin (5%), and wool fat (5%)	*Terminalia arjuna *(Combretaceae)	N	Sb	Fraction I hydroalcohol Fraction II phytoconstituents extraction of tannins Fraction III consisted of saponins	0.5 g	Diuretic, cooling, aphrodisiac, expectorant, antidysenteric, urinary astringent, antioxidant, and antibacterial activities	4 cm^2^	2/2 days	?	16 days

Swamy et al., 2006 [33]	India	Wistar rats	24	♂♀	?	150–200 g	4	6	?	Framycetin ointment	*Embelia ribes *(Myrsinaceae)	?	L	Embelin	4 mg/mL of 0.2% sodium alginate gel	Anti-inflammatory to relieve rheumatism and fever activities	500 mm^2^	4/4 days	?	16 days

Hernandes et al., 2010 [34]	Brazil	Wistar rats	15	♂	?	180–200 g	3	5	1	Ointment base	*Stryphnodendron adstringens *(Fabaceae)	?	Sb	EtOAc fraction	?	Antioxidant, cicatrizant, and anti-inflammatory activities	7 mm^2^	4, 7, and 10 days	?	10 days

Sidhu et al., 1999 [35]	USA and India	Sprague Dawley rats	?	♂	?	250–300 g	4	?	1	Vehicle PBS	*Arnebia nobilis *(Boraginaceae)	N	?	Arnebin-1 (5,8-dihydroxy-2-(19-b,b-dimethylaryoxy-49- methylpent-3-enyl)-1,4-naphthoquinone	?	?	8 mm^2^	Daily	?	11 days

Paramesha et al., 2015 [36]	India	Wistar rats	18	?	?	150–200 g	3	6	?	Sodium alginate	*Carthamus tinctorius *(Asteraceae)	N	L	Dehydroabietylamine of *C. tinctorius* L., var. Annigeri-2	50 g to get 0.2% (w/w) ointment gel	Laxative, appetizer, and diuretic also useful in urorrhea and ophthalmopathy activities	?	4/4 days	?	16 days

Nagappan et al., 2012 [37]	Malaysia	Sprague Dawley rats	84	♀	?	200–250 g	7	12	1	Not treated	*Murraya koenigii *(Rutaceae)	N	L	Carbazole alkaloids mahanine (1) (0.40%) (C_23_H_25_NO_2_), mahanimbicine (2) (0.24%) (C_23_H_25_NO), and mahanimbine (3) (0.66%)	Mahanine (1) (0.40%), (2) (0.24%), and (3) (0.66%) (w/w)	Stimulants, tonics, treating influenza, fever, and bronchial asthma activities	8 mm^2^	Daily	% of wound contraction = Ø of wound area − Ø of unhealed w.a./diameter of w.a. (wound area) × 100%	18 days

Qu et al., 2013 [38]	China	Sprague Dawley rats	54	♂	?	200–220 g	9	6	1	Vaseline	*Amorpha fruticosa *(Fabaceae)	N	Fr	6a,12a–dehydroamorphin, D–3–O–methyl–chiro–inositol, Kaempferol-3-gluco- 7-rhamnoside,7,2′,4′,5′-tetrom–ethoxyoflavone, dehydrosermundone, tephrosin, 7,4′-dimethoxyisoflavone	?	?	500 mm^2^	2/2 days	Percent wound contraction = (original wound area − unhealed area)/original wound area × 100%	22 days

**(b):** 

References	Country	Animal strain	Animal number	Sex	Age	Weight	Experimental groups	Animal number per group	Numbers of animals per box	Treatment control group	Plant species	Native/exotic	Used parts	Isolated	Dose	Popular indication	Wound area	Measurement interval	Wound area calculation	Treatment time
Ghosh et al., 2012 [39]	India	Wistar rats/Swiss albino mice	36	♂	?	150–180 g/20–25 g	6	6	?	Ointment base	*Pedilanthus tithymaloides* (Euphorbiaceae)	N	L	2-(3,4-Dihydroxy-phenyl)-5,7-dihydroxy-chromen-4-one, 1, 2-tetradecanediol, and 1-(hydrogen sulfate)	50 mg	Antiviral, antibacterial, antihemorrhagic, antitumor, abortive, and anti-inflammatory activities	500 mm^2^	3/3 days	(%) wound contraction 1/4 (initial × final wound area) × 100	21 days

Mukherjee et al., 2013 [40]	India	Swiss albino mice/Wistar rats	?	?	?	18–20 g/150–180 g	10	?	?	Ointment base and povidone iodine	*Shorea robusta* (Dipterocarpaceae)	N	L	Compound I: bioactive bergenin and compound II: triterpene ursolic acid	0.025 g of isolated compounds 1 and 2 mixed with 10 g ointment base	Wounds and burn healing	6 cm	3/3 days	Two-day interval/(wound area on day 0 × wound area on day *n*)/wound area on day 0 × 100	?

Melo et al., 2011 [41]	Brazil	Swiss mice	30	♀	12 wk	45.0 ± 2.0 g	4	?	1	NaCl	*Cratylia mollis* (Leguminoceae)	N	S	Cramoll 1,4 lectin	100 g/mL	?	0.5 cm	2, 7, and 12 days	*A* = *π* × *R* × *r*	12 days

Pieters et al., 1995 [20]	Belgium	Wistar rats	40	♀	?	250–300 g	20	2	1	Not treated	*Croton* spp. (Euphorbiaceae)	E	Lx	3′,4-0-Dimethylcedrusin, taspine hydrochloride	0,5 mL/2x day	Wound healing activities	3 cm	Daily	?	18 days

Ahamed et al., 2008 [42]	India	Wistar rats	24	♂♀	?	240–250 g	4	6	6	Tween 80	*Grewia tiliaefolia* (Tiliaceae)	N	Sb	Gulonic acid *γ*-lactone (GAGL)	50 mg/1x day	Burns, skin diseases, inflammation, diarrhea and pruritus, chronic wounds, and gastric ulcers	?	4/4 days	?	16 days

Zyuz'kov et al., 2012 [43]	Russian	?/mice	136	♂	2 mo	22–24 g	6	?	?	Water	*Aconitum baikalensis* (Ranunculaceae)	?	?	Songorine, napelline, hypaconitine, 12-epinapelline N-oxide, and mesaconitine	30 mL	?	10 × 10 mm	Daily	?	16 days

Singh et al., 2005 [44]	India	Wistar rats	20	♂♀	?	150–200 g	5	6	?	Tragacanth	*Elephantopus scaber* (Asteraceae)	N	L	Deoxyelephantopin	50 mg	Dysuria, diarrhea, dysentery, stomach pain; eczema and ulcers, and wound healing	500 mm^2^	4/4 days	?	14 days

Sharath et al., 2010 [45]	India	Wistar rats	?	♂♀	?	200–250 g	2	5	?	Nitrofurazone	*Bacopa monnieri* (Scrophulariaceae)	N	?	Bacoside-A	200 mg	Laxative, ulcers, anemia, leucoderma, and scabies	500 mm^2^	4/4 days	?	16 days

Vidya et al., 2012 [46]	India	Wistar rats	30	?	?	160–200 g	4	6	?	Nitrofurazone	*Entada pursaetha* (Mimosaceae)	N	S	Entadamide, phaseoloidin, and entagenic acid	?	Cancer, dropsy, eye diseases, wounds, snake bite, respiratory problems, and antibacterial	500 mm^2^	4/4 days	?	16 days

Ad: adults; wk: week; mo: month; d: days; ♂: male; ♀: female; N: native; E: exotic; L: leaves; F: flowers; Sb: stem bark; S: seed; Wp: whole plant; Fr: fruits; Lx: latex; ?: not related.

**(a):** 

Reference	Wound closure analysis	Reepithelialization analysis	Oxidative stress	Granulation tissue fill	Tensile strengths
Bastos et al., 2011	?	Fractions A and B: moderated 9 days Fractions A and B: 100% 15 days	?	After 15 days in the treated rats, the wound healing process by stimulating different biological events such as network of fibrin, epithelialization, granulation tissue, neovascularization, and wound contraction	?

Shukla et al., 1999	?	?	*Increased*: Superoxide dismutase (35%), catalase (67%), and glutathione peroxidase (49%) *Reduced*: Glutathione (17%)	?	?

Muralidhar et al., 2011	Petroleum ether fraction: (86.83 ± 0.87%) 16 days Benzene fraction: (86.67 ± 0.67%) 16 days Chloroform fraction: (88.0 ± 0.57%) 16 days Acetone fraction: (96.0 ± 0.37%) 16 days Control: (85.17 ± 0.79%) 16 days	*Epithelialization in days* Petroleum ether fraction: 21.17 ± 0.48% Benzene fraction: 21.67 ± 0.42% Chloroform fraction: 21.83 ± 0.48% Acetone fraction: 16.67 ± 0.42% Control: 22.0 ± 0.37%	?	*Hydroxyproline content (μg/mg)* Petroleum ether fraction: 21.57 ± 0.21 Benzene fraction: 20.96 ± 0.08 Chloroform fraction: 21.84 ± 0.08 Acetone fraction: 23.50 ± 0.17 Control: 21.48 ± 0.17	Petroleum ether fraction: 155.83 ± 2.26 g Benzene fraction: 151.0 ± 2.59 g Chloroform fraction: 163.33 ± 1.33 g Acetone fraction: 212.83 ± 2.02 g Control: 147.33 ± 1.23 g

Süntar et al., 2013	*Wound area (mm* ^*2*^ *) ± SEM (contraction%) in 12 days* Hg-MeOH: 0.96 ± 0.30 (65.71%) Hg-Hexane: 2.37 ± 0.11 (15.36%) Hg-CH_2_Cl_2_: 2.35 ± 0.29 (16.07%) Hg-EtOAc: 1.47 ± 0.32 (47.50%) Hg-BuOH: 1.74 ± 0.48 (37.86%) Hg-R-H_2_O: 2.63 ± 0.17 (6.07%) Hg-Fr.A: 2.20 ± 0.39 (20.29%) Hg-Fr.B: 1.65 ± 0.09 (40.22%) Hg-Fr.C: 1.83 ± 0.14 (33.69%) Control: 2.76 ± 0.30 (6.44%)	Tissues treated with Hg-MeOH, Hg-EtOAc, and Hg-Fr.B demonstrated good wound recovery with faster reepithelialization compared to the other groups tested	?	*Hydroxyproline content (μg/mg)* Hg-MeOH: 26.3 ± 1.0 Hg-Hexane: 18.5 ± 2.1 Hg-CH_2_Cl_2_: 19.7 ± 1.9 Hg-EtOAc: 31.2 ± 0.9 Hg-BuOH: 15.6 ± 1.8 Hg-R-H_2_O: 13.3 ± 1.8 Hg-Fr.A: 15.4 ± 1.2 Hg-Fr.B: 25.5 ± 1.2 Hg-Fr.C: 16.3 ± 1.9 Control: 8.9 ± 2.1	Hg-MeOH: 30.11% Hg-Hexane: 17.5% Hg-CH_2_Cl_2_: 15.2% Hg-EtOAc: 28.5% Hg-BuOH: 25.8% Hg-R-H_2_O: 11.6% Hg-Fr.A: 13.9% Hg-Fr.B: 25.2% Hg-Fr.C: 21.3% Control: 5.8%

Mekonnen et al. 2013	*Wound contraction in 12 days* Chloroform: xerogel: (77.517 ± 1.88), 5%: (79.91 ± 71.30), and 10%: (82.63 ± 1.74) Methanol: simple ointment: (86.21 ± 1.5), 5%: (90.86 ± 0.21), and 10%: (92.09 ± 2.00) Control: (96.63 ± 0.32)	*Epithelialization in days* Chloroform: xerogel: (17.83 ± 0.30), 5%: (17.16 ± 0.60), and 10%: (16.83 ± 0.65) Methanol: simple ointment: (17.33 ± 0.33), 5%: (15.66 ± 0.21), and 10%: (15.33 ± 0.66) Positive control: (14.00 ± 0.44)	?	*Hydroxyproline content (μg/mg)* Chloroform: xerogel: (3.01 ± 0.46), 5%: (5.83 ± 0.79), and 10%: (7.08 ± 2.08) Methanol: simple ointment: (3.29 ± 0.66), 5%: (11.01 ± 0.53), and 10%: (15.33 ± 0.66) Control: (12.57 ± 2.59)	Chloroform: xerogel: 190.83 ± 15.62 g (14.26%), 5%: 238.33 ± 22.86 g (24.89%), and 10%: 265.00 ± 33.04 g (38.86%) Methanol: simple ointment: 201.50 ± 10.05 g (20.65%), 5%: 322.00 ± 23.63 g (59.80%), and 10%: 336.83 ± 28.39 g (67.16%) Control: 402.33 ± 30.26 g

Pieters et al., 1995	PEG ointment: (70%) 15 days PEG 400 10%: (80%) 15 days Polyphenolic fraction from dragon's blood in H_2_O: (90%) 15 days Control: (60%) 15 days	PEG ointment: ++ (15 days) PEG 400 10%: ++ (15 days) Polyphenolic fraction from dragon's blood in H_2_O: ++ (15 days) Control: + (15 days)	?	*Crust presence* PEG ointment: after 4 days PEG 400 10%: after 5 days Polyphenolic fraction from dragon's blood in H_2_O: after 1 day Control: after 3 days	?

Korkina et al., 2007	Both verbascoside 56% (46,29 ± 12,21%) 8 days Both verbascoside 97% (124,29 ± 31,23%) 8 days Teupolioside 70% (78,39 ± 21,75%) 8 days Teupolioside 97% (98,45 ± 24,26%) 8 days Control (150,16 ± 65,46%) 8 days	?	*Lipid peroxidation* Both verbascoside 56% (7,4 ± 0,6%) Both verbascoside 97% (5,8 ± 0,4%) Teupolioside 70% (12,0 ± 0,7%) Teupolioside 97% (9,4 ± 0,6%) Control: (10,3 ± 1,0) *Glutathione (GST)* Both verbascoside 56% (3,0 ± 1,3%) Both verbascoside 97% (5,1 ± 1,3%) Teupolioside 70% (3,4 ± 1,3%) Teupolioside 97% (5,9 ± 1,2%) Control: (3,6 ± 1.3%) *Superoxide dismutases* Both verbascoside 56% (2,5 ± 0,1%) Both verbascoside 97% (2,2 ± 0,1%) Teupolioside 70% (3,1 ± 0,3%) Teupolioside 97% (1,0 ± 0,1%) Control: (4,5 ± 0,5%)	?	?

Bigoniya et al., 2013	EHTF 200 (71,01 ± 4,25%) 16 days EHTF 400 (69,98 ± 3,34%) 16 days EHTF 600 (6,02 ± 0,79%) 16 days Control (71,65 ± 3,21%) 16 days	EHTF 200 (19,66 ± 2,85%) EHTF 400 (19,50 ± 2,63%) EHTF 600 (17,50 ± 1,56%) Control (21,50 ± 1,22%)	*Vehicle control*: catalase (0,46 ± 0,02%); SOD (1,15 ± 0,12%), and total protein (2,60 ± 0,06%) *EHTF 200*: catalase (0,45 ± 0,03%), SOD (1,16 ± 0,06%), and total protein (2,69 ± 0,07%) *EHTF 400*: catalase (0,52 ± 0,09%), SOD (2,63 ± 0,15%), and total protein (3,34 ± 0,05%) *EHTF 600*: catalase (0,75 ± 0,19%), SOD (5,06 ± 0,09%), and total protein (4,02 ± 0,03%)	*Hydroxyproline content* EHTF 200 (15,89 ± 1,28%) EHTF 400 (17,89 ± 2,26%) EHTF 600 (24,14 ± 2,23%) Control (16,09 ± 1,35%)	?

Lodhi et al., 2011	MAF A (100,00%) 20 days MAF B (100,00%) 20 days MAF C (100,00%) 18 days Control (90,37 ± 2,07%) 20 days	MAF A and B (20 days) MAF C (18 days) Control (24 days)	?	*Hydroxyproline content*: MAF A (37,11 ± 1,25%) MAF B (32,86 ± 0,85%) MAF C (42,01 ± 0,82%) Control (21,74 ± 1,85%) *Protein content* MAF A (56,30 ± 0,55%) MAF B (52,50 ± 1,70%) MAF C (83,60 ± 0,72%) Control (47,30 ± 1,72%)	MAF A (603,00 ± 12,01%) MAF B (635,00 ± 9,68%) MAF C (850,00 ± 11,89%) Control (423,00 ± 10,96%)

Tabandeh et al., 2013	Silibinin 10%: 100% (18 days) Silibinin 20%: 100% (22 days) Control: 100% (26 days)	?	?	*Content N-acetyl glucosamine and n-acetyl galactosamine*: silibinin 10 and 20% ↑ compared with the control groups at days 10, 20, and 30 *Hydroxyproline and collagen content*: silibinin 10 and 20% ↑ compared with the control groups at days 10, 20, and 30	?

Sonmez et al., 2015	Absorbable polysaccharide haemostat (APH): (94.74 ± 0.02%) 14 days Control: 87.33 ± 0.02% 14 days	?	?	*Type 1 collagen* APH: 4.25 Control: 3.25 *Fibroblast density* APH: 2.87 Control: 1.75	?

Karakaş et al., 2012	HOT: (80%) 30 days HOTBp: (100%) 30 days Control: (80%) 30 days	?	?	HOT: ↑ fibroblastic and lymphocytes: 5 days HOTBp: ↑ fibroblastic and lymphocytes: 5 days Control: ↑ fibroblastic and lymphocytes: 5 days HOT: ↑ collagen fibrils: 10 days HOTBp: ↑ collagen fibrils: 10 days Control: ↑ collagen fibrils: 10 days	?

Choi et al., 2001	G1G1M1DI2: (98,9%) 8 days Control: (69,5%) 8 days	*Epithelialization in 8 days* G1G1M1DI2: 98,9% Control: 69,5%	?	*EGF receptor* G1G1M1DL2 0,5%: (113%) G1G1M1DL2 50%: (220 ± 8%) Control: 100% *Fibronectin* G1G1M1DL2 0,5%: (294 ± 34%) G1G1M1DL2 50%: (408 ± 80%) Control: 100% *Fibronectin receptor* G1G1M1DL2 0,5%: (159 ± 11%) G1G1M1DL2 50%: (220 ± 19%) Control: 100%	?

Parente et al., 2011	?	**?**	?	Number of blood vessels HCF 1 (0/4) DCF 2 (0/13) Control 2 (0/13) Days 4 and 7: presence of fibrin in both groups	?

Olugbuyiro et al., 2010	*Flabellaria paniculata* Chloroform fraction: 0.0 (100%) 14 days Aqueous fraction: 25.0 ± 3.0% (71.4%) 14 days Control: 87.5 ± 7.5%	*Flabellaria paniculata on noninfected rat wounds* Chloroform fraction: (14.0 ± 0.0%) Aqueous fraction: (21.5 ± 0.5%) Control: (24.5 ± 0.5%)	?	?	?

Süntar et al., 2010	mm^2^ (%) Fr.A: 1.60 ± 1.53 (44.4%) Fr.B: 1.59 ± 0.11 (44.8%) Fr.C: 0.99 ± 0.31 (65.6%) Fr.D: 0.77 ± 0.03 (73.3%) Fr.E: 1.98 ± 0.63 (31.3%) Control: 2.88 ± 0.72 (17.5%)	?	?	**?**	mm^2^ (%) Fr.A: 21.52 ± 1.15 (13.9%) Fr.B: 24.97 ± 3.18 (32.3%) Fr.C: 25.63 ± 1.43 (35.8%) Fr.D: 26.61 ± 2.05 (40.9%) Fr.E: 22.95 ± 2.73 (21.6%) Control: 18.88 ± 2.67 (16.3%)

Kim et al., 2013	The ginsenoside Rd-treated wounds were significantly smaller than the wounds treated with control Matrigel on days 6 and 9	?	?	Ginsenoside Rd ↑ proliferation and migration fibroblasts; ginsenoside Rd at 0.1–10 mM ↑ collagen type I protein and ↓ MMP-1 protein in fibroblasts	?

Chaudhari et al., 2006	?	Fraction I: 9 days Fraction II: 23 days Fraction III: 20 days	?	Fraction I increase in hexosamine Fractions II and III did not reveal increase in the hexosamine content of granulation tissue	Fraction I: 719.33 g ± 0.88 Fraction II: 572.33 g ± 2.46 Fraction III: 590.33 g ± 1.87

Swamy et al., 2006	Embelin: (98.50% ± 1.64) 16 days Control: (85.33% ± 3.66) 16 days	*Epithelialization in days* Embelin: 18.17 ± 1.47 Control: 20.33 ± 2.66	?	Granulation tissue showed complete healing with more fibroblasts, collagen, and increased number of blood vessels	Embelin: 528.00 g ± 15.85 Control: 374.67 g ± 5564

Hernandes et al., 2010	The 1% ethyl-acetate fraction from *Stryphnodendron adstringens* did not influence wound contraction	No difference in the length of newly formed epithelium was found between the treated and control wounds	?	?	?

**(b):** 

Reference	Wound closure analysis	Reepithelialization analysis	Oxidative stress	Granulation tissue fill	Tensile strengths
Sidhu et al., 1999	Arnebin-1 reduced wound width wounds compared with control	Arnebin-1: 7 days Control: only epithelial migration over the dermis	?	The organization of the granulation tissue was more advanced in arnebin-1-treated wounds with thick bundles of well-aligned collagen compared with controls	?

Paramesha et al., 2015	Dehydroabietylamine: (97.78% ± 2.15) 16 days Control: (82.92% ± 1.83) 16 days	*Epithelialization in days* Dehydroabietylamine: 17.67 ± 2.62 Control: 23.17 ± 1.14	?	*Hydroxyproline content (µg/100 g)* Dehydroabietylamine: 2106,50 ± 2,62 Control: 1369,67 ± 10,54	Dehydroabietylamine: 425.67 g ± 10.03 Control: 277.00 g ± 9.39

Nagappan et al., 2012	Mahanine and mahanimbicine: (88.5% ± 2.03 to 93% ± 2.04) 16 days Control: (82.7% ± 2.13) 16 days	Mahanine and mahanimbicine: 18 days Control: 18 days	?	*Collagen deposition* Mahanine and mahanimbicine: (65.63% ± 0.87 to 67.76% ± 0.85) 21 days and (81.56% ± 1.04 to 88.54% ± 1.34) 28 days Control: (61.84% ± 0.94) 21 days and (78.06% ± 1.22) 28 days	?

Qu et al., 2013	Compound I to compound VII: (96.8% ± 1.9 to 87.0% ± 2.6) 16 days Control: (87.2% ± 3.1) 16 days	Compound I and compound V: 18 days Control and other groups: 22 days	?	*Hydroxyproline content (mg/g tissue)* Compound I to compound VII: 58.4 ± 3.7 to 80.3 ± 4.4 Control: 60.2 ± 4.1	Compound I to compound VII: 431.5 g ± 8.3 to 547.3 g ± 7.9 Control: 436.5 g ± 7.6

Ghosh et al., 2012	Compound I to compound II: (100%) 18 days Control: (100%) 22 days	Compound I: 17.16 ± 0.4 days Compound II: 17.25 ± 0.25 days Control: 22.00 ± 0.1 days	?	Compounds I and II: fibrous connective tissue with strong collagenation Control: fibrosis and more aggregation of macrophages with less collagen fibers	Compound I: 565.10 g ± 3.1 Compound II: 561.12 g ± 3.9 Control: 372.13 g ± 3.23

Mukherjee et al., 2013	Compound I (2,5%): (89.91% ± 0.55) 18 days Compound II (2,5%): (97.89% ± 0.77) 18 days Control: (75.44% ± 0.37) 18 days	Compound I (2,5%): 17.16 ± 0.4 days Compound II (2,5%): 16.01 ± 0.33 days Control: 21.00 ± 0.11 days	?	*Hydroxyproline content (mg/g tissue)* Compound I (2,5%): 158.23 ± 0.44 Compound II (2,5%): 198.16 ± 0.33 Control: 151.9 ± 2.69	Compound I (2,5%): 538.00 g ± 1.89 Compound II (2,5%): 535.12 g ± 3.59 Control: 322.39 g ± 2.66

Melo et al., 2011	Cramoll 1,4: (100%) 10 days Control: (100%) 12 days	?	?	*Crust presence*: cramoll 1,4: 13.1 ± 7.02 Control: 5.4 ± 3.3 *Collagen presence*: cramoll 1,4: (higher collagen deposition and annex sprouts) 12 days Control: (matrix poor in collagen fibers) 12 days	?

Pieters et al., 1995	3′,4-0-Dimethylcedrusin: (85%) 15 days Taspine: (75%) 15 days Control: (60%) 15 days	3′,4-0-Dimethylcedrusin: ++ (15 days) Taspine: + (15 days) Control: + (15 days)	?	*Crust presence* 3′,4-0-Dimethylcedrusin: after 5 days Taspine: after 5 days Control: after 3 days	?

Ahamed et al., 2009	Gulonic acid *γ*-lactone: (94.02% ± 0.20) 16 days Control: (79.53% ± 0.97) 16 days	*Epithelialization in days* Gulonic acid *γ*-lactone: 18.62 ± 0.21 Control: 22.59 ± 0.15	?	*Hydroxyproline content (µg/100 g)* Gulonic acid *γ*-lactone: 780.48 ± 50.73 Control: 346.15 ± 14.54 *Fibroblast count/high power field × 400* Gulonic acid *γ*-lactone: 53.26 ± 2.37 Control: 97.53 ± 4.26 *Blood vessel count/high power field × 400* Gulonic acid *γ*-lactone: 21.94 ± 1.15 Control: 11.63 ± 1.11	Gulonic acid *γ*-lactone: 561.12 g ± 5.18 Control: 327.63 g ± 6.37

Zyuz'kov et al., 2012	Songorine: 100% (9–16 days) Napelline: 100% (9–16 days) Hypaconitine: 100% (9–16 days) 12-Epinapelline N-oxide: 89.93% ± 5.53 (9–16 days) Mesaconitine: 97.8% ± 2.2 (9–16 days) Control: 89.72% ± 4.72 (9–16 days)	Songorine-napelline-hypaconitine Newly formed epithelium by the wound edges represented a cell layer of varying thickness without vertical anisomorphism: 5 days	?	*Leukocytic infiltration* Songorine: reduction (3 days) Napelline: reduction (3 days) Hypaconitine: reduction (3 days) 12-Epinapelline N-oxide: ?/mesaconitine: ?/control: ? *Counts of fibroblasts* Songorine: increased (3 days) Napelline: increased (3 days) Hypaconitine: increased (3 days) 12-Epinapelline N-oxide: ?/mesaconitine: ?/control: ?	?

Singh et al., 2005	Deoxyelephantopin: 98.8% ± 0.35 (16 days) Control: 85.8% ± 0.69 (16 days)	*Epithelialization in days* Deoxyelephantopin: 14.0 ± 0.26 Control: 20.0 ± 0.86	?	Deoxyelephantopin: ↓ macrophages and ↑ collagen formation Control: ↑ macrophages and ↓ collagen formation	Deoxyelephantopin: 412.0 g ± 11.37 Control: 298.6 g ± 8.48

Sharath et al., 2010	Bacoside-A: 98.18% ± 0.05 (16 days) Control: 85.22% ± 0.02 (16 days)	*Epithelialization in days* Bacoside-A: 18.30 ± 0.01 Control: 20.20 ± 0.04	?	Bacoside-A: ↑ blood vessels and ↑ collagen formation Control: ↑ inflammatory cells, ↓ blood vessels, and ↓ collagen formation	Bacoside-A: 538.47 g ± 0.14 Control: 380.48 g ± 0.11

Vidya et al., 2012	Entadamide: 92.22% ± 0.05 (16 days) Phaseoloidin: 88.50 ± 0.10 (16 days) Entagenic acid: 96.08% ± 0.04 (16 days) Control: 83.31% ± 1.06 (16 days)	*Epithelialization in days* Entadamide: 19.92 ± 0.01 Phaseoloidin: 21.16 ± 0.02 Entagenic acid: 18.08 ± 0.01 Control: 24.00 ± 0	?	*Hydroxyproline content (µg/100 g)* Entadamide: 1891.17 ± 2.75 Phaseoloidin: 1690.33 ± 2.80 Entagenic acid: 2001.33 ± 3.53 Control: 1369.67 ± 10.54	Entadamide: 463.33 g ± 4.48 Phaseoloidin: 450.17 g ± 7.55 Entagenic acid: 549.83 g ± 2.21 Control: 260.83 g ± 14.05
