# Supplementary material for: Effect of Topical Administration of Fractions and Isolated Molecules from Plant Extracts on Skin Wound Healing: A Systematic Review of Murine Experimental Models
Source: Mediators Inflamm. 2016 Oct 17;2016:4916068. doi: 10.1155/2016/4916068 (PMC5086515; doi:10.1155/2016/4916068)
Supplement: Supplementary file 1 — The full search strategy is presented in Table S1. Keywords were obtained in the MeSH (Medical Subject Headings) database, which is the U.S. National Library of Medicine (NLM) controlled vocabulary thesaurus used for indexing manuscripts in PubMed. In the additional databases used to recover relevant articles, the keywords were adapted acording the search algoritm adopted in the search plataforms. Table S2 shows the results of bias analysis. All criteria investigated were based on ARRIVE guideline, which states the essential elements that should be reported in In Vivo animal experiments. [file 4916068.f1.pdf]

Table S1: Full search strategy in PubMed and Scopus, including search terms and filters.

Data base	Descriptors	Items Found	Time	Date
PubMed	Animals: #1 OR #2<br><br>#1 Filters<br><br>("animal experimentation"[MeSH Terms] OR "models, animal"[MeSH Terms] OR "invertebrates"[MeSH Terms] OR "Animals"[Mesh:noexp] OR "animal population groups"[MeSH Terms] OR "chordata"[MeSH Terms:noexp] OR "chordata, nonvertebrate"[MeSH Terms] OR "vertebrates"[MeSH Terms:noexp] OR "amphibians"[MeSH Terms] OR "birds"[MeSH Terms] OR "fishes"[MeSH Terms] OR "reptiles"[MeSH Terms] OR "mammals"[MeSH Terms:noexp] OR "primates"[MeSH Terms:noexp] OR "artiodactyla"[MeSH Terms] OR "carnivora"[MeSH Terms] OR "cetacea"[MeSH Terms] OR "chiroptera"[MeSH Terms] OR "elephants"[MeSH Terms] OR "hyraxes"[MeSH Terms] OR "insectivora"[MeSH Terms] OR "lagomorpha"[MeSH Terms] OR "marsupialia"[MeSH Terms] OR "monotremata"[MeSH Terms] OR "perissodactyla"[MeSH Terms] OR "rodentia"[MeSH Terms] OR "scandentia"[MeSH Terms] OR "sirenia"[MeSH Terms] OR "xenarthra"[MeSH Terms] OR "haplorhini"[MeSH Terms:noexp] OR "strepsirhini"[MeSH Terms] OR "platyrrhini"[MeSH Terms] OR "tarsii"[MeSH Terms] OR "catarrhini"[MeSH Terms:noexp] OR "cercopithecidae"[MeSH Terms] OR "hylobatidae"[MeSH Terms] OR "hominidae"[MeSH Terms:noexp] OR "gorilla gorilla"[MeSH Terms] OR "pan paniscus"[MeSH Terms] OR "pan troglodytes"[MeSH Terms] OR "pongo pygmaeus"[MeSH Terms])<br><br>#2 Filters<br><br>((animals[tiab] OR animal[tiab] OR mice[Tiab] OR mus[Tiab] OR mouse[Tiab] OR murine[Tiab] OR woodmouse[tiab] OR rats[Tiab] OR rat[Tiab] OR murinae[Tiab] OR muridae[Tiab] OR cottonrat[tiab] OR cottonrats[tiab] OR hamster[tiab] OR hamsters[tiab] OR cricetinae[tiab] OR rodentia[Tiab] OR rodent[Tiab] OR rodents[Tiab] OR pigs[Tiab] OR pig[Tiab] OR swine[tiab] OR swines[tiab] OR piglets[tiab] OR piglet[tiab] OR boar[tiab] OR boars[tiab] OR "sus scrofa"[tiab] OR ferrets[tiab] OR ferret[tiab] OR polecat[tiab] OR polecats[tiab] OR "mustela putorius"[tiab] OR "guinea pigs"[Tiab] OR "guinea pig"[Tiab] OR cavia[Tiab] OR callithrix[Tiab] OR marmoset[Tiab] OR marmosets[Tiab] OR cebuella[Tiab] OR hapale[Tiab] OR octodon[Tiab] OR chinchilla[Tiab] OR chinchillas[Tiab] OR gerbillinae[Tiab] OR gerbil[Tiab] OR gerbils[Tiab] OR jird[Tiab] OR jirds[Tiab] OR merione[Tiab] OR meriones[Tiab] OR rabbits[Tiab] OR rabbit[Tiab] OR hares[Tiab] OR hare[Tiab] OR diptera[Tiab] OR flies[Tiab] OR fly[Tiab] OR dipteral[Tiab] OR drosphila[Tiab] OR drosophilidae[Tiab] OR cats[Tiab] OR cat[Tiab] OR carus[Tiab] OR felis[Tiab] OR nematoda[Tiab] OR nematode[Tiab] OR nematoda[Tiab] OR nematode[Tiab] OR nematodes[Tiab] OR sipunculida[Tiab] OR dogs[Tiab] OR dog[Tiab] OR canine[Tiab] OR canines[Tiab] OR canis[Tiab] OR sheep[Tiab] OR sheeps[Tiab] OR mouflon[Tiab] OR mouflons[Tiab] OR ovis[Tiab] OR goats[Tiab] OR goat[Tiab] OR capra[Tiab] OR capras[Tiab] OR rupicapra[Tiab] OR chamois[Tiab] OR haplorhini[Tiab] OR monkey[Tiab] OR monkeys[Tiab] OR anthropoidea[Tiab] OR anthropoids[Tiab] OR saguinus[Tiab] OR tamarin[Tiab] OR tamarins[Tiab] OR leontopithecus[Tiab] OR hominidae[Tiab] OR ape[Tiab] OR apes[Tiab] OR pan[Tiab] OR paniscus[Tiab] OR "pan paniscus"[Tiab] OR bonobo[Tiab] OR bonobos[Tiab] OR troglodytes[Tiab] OR "pan troglodytes"[Tiab] OR gibbon[Tiab] OR gibbons[Tiab] OR siamang[Tiab] OR siamangs[Tiab] OR nomascus[Tiab] OR symphalangus[Tiab] OR chimpanzee[Tiab] OR chimpanzees[Tiab] OR prosimians[Tiab] OR "bush baby"[Tiab] OR prosimian[Tiab] OR bush babies[Tiab] OR galagos[Tiab] OR galago[Tiab] OR pongidae[Tiab] OR gorilla[Tiab] OR gorillas[Tiab] OR pongo[Tiab] OR pygmaeus[Tiab] OR "pongo pygmaeus"[Tiab] OR orangutans[Tiab] OR pygmaeus[Tiab] OR lemur[Tiab] OR lemurs[Tiab] OR lemuridae[Tiab] OR horse[Tiab] OR horses[Tiab] OR pongo[Tiab] OR equus[Tiab] OR cow[Tiab] OR calf[Tiab] OR bull[Tiab] OR chicken[Tiab] OR chickens[Tiab] OR gallus[Tiab] OR quail[Tiab] OR bird[Tiab] OR birds[Tiab] OR quails[Tiab] OR poultry[Tiab] OR poultries[Tiab] OR fowl[Tiab] OR fowls[Tiab] OR reptile[Tiab] OR reptilia[Tiab] OR reptiles[Tiab] OR snakes[Tiab] OR snake[Tiab] OR lizard[Tiab] OR lizards[Tiab] OR alligator[Tiab] OR alligators[Tiab] OR crocodile[Tiab] OR crocodiles[Tiab] OR turtle[Tiab] OR turtles[Tiab] OR amphibian[Tiab] OR amphibians[Tiab] OR amphibia[Tiab] OR frog[Tiab] OR frogs[Tiab] OR bombina[Tiab] OR salientia[Tiab] OR toad[Tiab] OR toads[Tiab] OR "epidalea calamita"[Tiab] OR salamander[Tiab] OR salamanders[Tiab] OR eel[Tiab] OR eels[Tiab] OR fish[Tiab] OR fishes[Tiab] OR pisces[Tiab] OR catfish[Tiab] OR catfishes[Tiab] OR siluriformes[Tiab] OR arius[Tiab] OR heteropneustes[Tiab] OR sheatfish[Tiab] OR perch[Tiab] OR perches[Tiab] OR percidae[Tiab] OR perca[Tiab] OR trout[Tiab] OR trouts[Tiab] OR char[Tiab] OR chars[Tiab] OR salvelinus[Tiab] OR "fathead minnow"[Tiab] OR minnow[Tiab] OR cyprinidae[Tiab] OR carps[Tiab] OR carp[Tiab] OR zebrafish[Tiab] OR zebrafishes[Tiab] OR goldfish[Tiab] OR goldfishes[Tiab] OR guppy[Tiab] OR guppies[Tiab] OR chub[Tiab] OR chubs[Tiab] OR tinca[Tiab] OR barbels[Tiab] OR barbus[Tiab] OR pimephales[Tiab] OR promelas[Tiab] OR "poecilia reticulata"[Tiab] OR mullet[Tiab] OR mullets[Tiab] OR seahorse[Tiab] OR seahorses[Tiab] OR mugil curema[Tiab] OR atlantic cod[Tiab] OR shark[Tiab] OR sharks[Tiab] OR catshark[Tiab] OR anguilla[Tiab] OR salmonid[Tiab] OR salmonids[Tiab] OR whitefish[Tiab] OR whitefishes[Tiab] OR salmon[Tiab] OR salmons[Tiab] OR sole[Tiab] OR solea[Tiab] OR "sea lamprey"[Tiab] OR lamprey[Tiab] OR lampreys[Tiab] OR pumpkinseed[Tiab] OR sunfish[Tiab] OR sunfishes[Tiab] OR tilapia[Tiab] OR tilapias[Tiab] OR turbot[Tiab] OR turbots[Tiab] OR flatfish[Tiab] OR flatfishes[Tiab] OR sciuridae[Tiab] OR squirrel[Tiab] OR squirrels[Tiab] OR chipmunk[Tiab] OR chipmunks[Tiab] OR suslik[Tiab] OR susliks[Tiab] OR vole[Tiab] OR voles[Tiab] OR lemming[Tiab] OR lemmings[Tiab] OR muskrat[Tiab] OR muskrats[Tiab]	5866942	12:40:07	04/09/2015

	OR lemmus[Tiab] OR otter[Tiab] OR otters[Tiab] OR marten[Tiab] OR martens[Tiab] OR martes[Tiab] OR weasel[Tiab] OR badger[Tiab] OR badgers[Tiab] OR ermine[Tiab] OR mink[Tiab] OR minks[Tiab] OR sable[Tiab] OR sables[Tiab] OR gulo[Tiab] OR gulos[Tiab] OR wolverine[Tiab] OR wolverines[Tiab] OR minks[Tiab] OR mustela[Tiab] OR llama[Tiab] OR llamas[Tiab] OR alpaca[Tiab] OR alpacas[Tiab] OR camelid[Tiab] OR camelids[Tiab] OR guanaco[Tiab] OR guanacos[Tiab] OR chiroptera[Tiab] OR chiropteras[Tiab] OR bat[Tiab] OR bats[Tiab] OR fox[Tiab] OR foxes[Tiab] OR iguana[Tiab] OR iguanas[Tiab] OR xenopus laevis[Tiab] OR parakeet[Tiab] OR parakeets[Tiab] OR parrot[Tiab] OR parrots[Tiab] OR donkey[Tiab] OR donkeys[Tiab] OR mule[Tiab] OR mules[Tiab] OR zebra[Tiab] OR zebras[Tiab] OR shrew[Tiab] OR shrews[Tiab] OR bison[Tiab] OR bisons[Tiab] OR buffalo[Tiab] OR buffaloes[Tiab] OR deer[Tiab] OR deers[Tiab] OR bear[Tiab] OR bears[Tiab] OR panda[Tiab] OR pandas[Tiab] OR "wild hog"[Tiab] OR "wild boar"[Tiab] OR fitchew[Tiab] OR fitch[Tiab] OR beaver[Tiab] OR beavers[Tiab] OR jerboa[Tiab] OR jerboas[Tiab] OR capybara[Tiab] OR capybaras[Tiab]) NOT medline[subset])			
	Plants: #3<br><br>#3 Filters<br><br>(“Plants, Medicinal”[MeSH Terms] OR “Plant Extracts”[MeSH Terms] OR “Phytotherapy”[MeSH Terms] OR “Herbal Therapy”[TIAB] OR “Herb Therapy”[TIAB] OR “Drugs, Chinese Herbal”[MeSH Terms] OR “Chinese Plant Extracts”[TIAB] OR “Herbal Drug”[TIAB] OR “Herbal extract”[TIAB] OR “Plant Preparations”[MeSH Terms] OR “Herbal Preparation”[TIAB] OR “Plant Bark”[MeSH Terms] OR “plant leaves”[MeSH Terms] OR “plant leaf”[TIAB] OR “seeds extract”[TIAB] OR “bark extract”[TIAB] OR “leaves extract”[TIAB] OR “root extract”[TIAB] OR “fruits extract”[TIAB] OR “flowers Extract”[TIAB] OR “Plant Oils”[MeSH Terms] OR “Vegetable Oils”[TIAB])	238064	12:37:50	04/09/2015
	Wound #4<br><br>#4 Filters<br><br>(“Wound Healing”[MeSH terms] OR “Regeneration”[MeSH terms])	174687	12:38:41	0409/2015
	Skin #5<br><br>#5 Filters<br><br>(“Skin”[MeSH terms] OR “Dermis”[MeSH terms] OR “Granulation Tissue”[MeSH terms] OR “Epidermis”[MeSH terms] OR “Keratinocytes”[MeSH terms] OR “Integumentary System”[MeSH terms] OR “Dermatology”[MeSH terms] OR “Dermoscopy”[MeSH terms] OR “Wounds and Injuries”[MeSH terms] OR “Fibrosis”[MeSH terms] OR “Skin injuries”[TIAB] OR “Skin fibrosis”[TIAB] OR “Skin scars”[TIAB] OR “Skin cicatriz”[TIAB] OR “Cicatrix”[MeSH terms])	1008716	12:38:56	04/09/2015
	**Total: #1OR#2 and #3 and #4 and #5**	**476**	**12:40:50**	**04/09/2015**
**Data base**	**Descriptors**	**Items Found**	**Time**	**Date**
**S c o p u s**	Plants: #1<br><br>#1 Filters<br><br>(TITLE-ABS-KEY(“Plants, Medicinal”) OR TITLE-ABS-KEY(“Plant Extracts”) OR TITLE-ABS-KEY(“Phytotherapy”) OR TITLE-ABS-KEY(“Herbal Therapy”) OR TITLE-ABS-KEY(“Herb Therapy”) OR TITLE-ABS-KEY(“Drugs, Chinese Herbal”) OR TITLE-ABS-KEY(“Chinese Plant Extracts”) OR TITLE-ABS-KEY(“Herbal Drug”) OR TITLE-ABS-KEY(“Herbal extract”) OR TITLE-ABS-KEY(“Plant Preparations”) OR TITLE-ABS-KEY(“Herbal Preparation”) OR TITLE-ABS-KEY(“Plant Bark”) OR TITLE-ABS-KEY(“plant leaves”) OR TITLE-ABS-KEY(“plant leaf”) OR TITLE-ABS-KEY(“seeds extract”) OR TITLE-ABS-KEY(“bark extract”) OR TITLE-ABS-KEY(“leaves extract”) OR TITLE-ABS-KEY(“root extract”) OR TITLE-ABS-KEY(“fruits extract”) OR TITLE-ABS-KEY(“flowers Extract”) OR TITLE-ABS-KEY(“Plant Oils”) OR TITLE-ABS-KEY(“Vegetable Oils”))	273.736	14:18:34	04/09/2015
	Wound: #2<br><br>#2 Filters<br><br>(TITLE-ABS-KEY(“Wound Healing”) OR TITLE-ABS-KEY(Regeneration) OR TITLE-ABS-KEY(repair))	686.423	14:27:33	04/09/2015

	Skin: #3<br><br>#3 Filters<br><br>(TITLE-ABS-KEY(Skin) OR TITLE-ABS-KEY(Dermis) OR TITLE-ABS-KEY("Granulation Tissue") OR TITLE-ABS-KEY(Epidermis) OR TITLE-ABS-KEY(Keratinocyte*) OR TITLE-ABS-KEY(Integumentary System) OR TITLE-ABS-KEY(Dermatology) OR TITLE-ABS-KEY(Dermoscopy) OR TITLE-ABS-KEY(Skin wounds) OR TITLE-ABS-KEY(Skin injuries) OR TITLE-ABS-KEY(Skin fibrosis) OR TITLE-ABS-KEY(Skin scar*) OR (Skin cicatrix))	1.057.572	14:44:23	04/09/2015
	Animals: #4<br><br>#4 Filters<br><br>Scopus gives us the option to limit the Experimental Animal research on keywords			
	**Total: #1 and #2 and #3 and #4**	**531**	**15:05:23**	**04/09/2015**

Table S2. Analysis of reporting bias (ARRIVE) in all included studies.

References	Bastos, et al., 2011	Shukla, et al., 1999	Muralidhar, et al., 2011	Süntar, et al., 2013	Mekonnen, et al. 2013	Pieters, et al., 1995	Korkina, et al., 2007	Bigoniya, et al., 2013	Lodhi, et al., 2011	Tabandeh, et al., 2013	Sonmez, et al., 2015	Karakaş, et al., 2012	Choi, et al., 2001	Parente, et al., 2011	Olugbuyiro, et al., 2010	Süntar, et al., 2010	Kim, et al., 2013	Chaudhari, et al., 2006	Swamy, et al., 2006	Hernandes, et al., 2010	Sidhu, et al., 1999	Paramesha, et al., 2015	Nagappan, et al., 2012	Qu, et al., 2013	Ghosh, et al., 2012	Mukherjee, et al., 2013	Melo, et al., 2011	Ahamed, et al., 2008	Zyuz'kov, et al., 2012	Singh, et al., 2005	Sharath, et al., 2010	Vidya, et al., 2012	
Accurate and concise description of the content of the article		✓	✓	✓	✓	✓		✓	✓	✓		✓		✓		✓	✓		✓	✓		✓	✓	✓	✓	✓	✓	✓	✓	✓	✓	✓	78.12%
**Abstract**																																	
Summary of the background, research objectives, methods, main findings, and conclusions	✓	✓	✓	✓	✓		✓	✓	✓	✓	✓	✓	✓	✓	✓	✓	✓	✓	✓	✓		✓	✓	✓	✓	✓	✓	✓	✓		✓	✓	90.6%
**Introduction**																																	
Sufficient scientific background			✓	✓	✓	✓	✓	✓		✓		✓	✓	✓	✓	✓		✓	✓	✓		✓	✓		✓	✓	✓	✓		✓	✓	✓	75%
Explanation of the experimetal approach and rationale	✓	✓				✓	✓	✓	✓	✓		✓		✓	✓			✓		✓	✓		✓	✓	✓		✓	✓			✓		59.37%
**Objectives**																																	
Clear primary and second objectives	✓		✓	✓	✓		✓	✓	✓	✓		✓	✓			✓	✓	✓	✓		✓		✓	✓	✓	✓	✓			✓	✓		68.75%
Material and methods																																	
Declaration Echical																																	
Nature of the ethical review permissions, relevant licneces and national or institutional guidelines for the care and use of animals	✓	✓	✓	✓	✓	✓	✓	✓	✓	✓	✓	✓	✓	✓	✓	✓	✓	✓	✓	✓	✓	✓	✓	✓	✓	✓	✓	✓	✓	✓	✓	✓	100.00%
**Study design**																																	
Number of animals per group experimental	✓		✓	✓	✓	✓	✓	✓	✓	✓	✓	✓	✓	✓	✓	✓	✓	✓	✓	✓		✓	✓	✓	✓			✓		✓	✓	✓	84.37%
Information on whether the experimental was performed as a blind controlled study																																	0.0%
**Experimental procedures**																																	
**Treatment**																																	
Dosage of treatment	✓	✓	✓	✓	✓	✓	✓			✓	✓	✓	✓	✓	✓	✓	✓	✓	✓	✓		✓	✓		✓	✓	✓	✓	✓	✓	✓	✓	87.5%
Route of administration	✓	✓	✓	✓	✓	✓	✓	✓	✓	✓	✓	✓	✓	✓	✓	✓		✓	✓	✓	✓		✓	✓	✓	✓	✓		✓	✓	✓	✓	90.62%
Duration of treatment	✓	✓	✓	✓	✓	✓	✓	✓	✓	✓	✓	✓	✓	✓	✓	✓	✓	✓	✓	✓	✓	✓	✓	✓	✓		✓	✓	✓	✓	✓	✓	96.87%
Time of day for treatment administration	✓		✓	✓	✓		✓	✓	✓	✓		✓	✓			✓	✓	✓	✓		✓		✓	✓	✓	✓	✓			✓	✓		68.75%
Location used for administration of treatment																																	0.0%
Rationale for choice of specific dosage				✓		✓								✓															✓				12.5%
Rationale for choice of specific route of administration																																	0.0%
**Experimental animals**																																	
Information regarding on animal species	✓	✓	✓	✓	✓	✓	✓	✓	✓	✓	✓	✓	✓	✓	✓	✓	✓	✓	✓	✓	✓	✓	✓	✓	✓	✓	✓	✓	✓	✓	✓	✓	100.0%
Strain of the animals	✓	✓	✓	✓	✓	✓	✓	✓	✓	✓	✓	✓	✓	✓	✓	✓	✓	✓	✓	✓	✓	✓	✓	✓	✓	✓	✓	✓		✓	✓	✓	96.87%
Sex of the animals	✓	✓		✓		✓	✓	✓	✓	✓	✓	✓	✓	✓	✓	✓	✓	✓	✓	✓	✓		✓	✓	✓		✓	✓	✓	✓	✓		84.37%
Animals weigth range	✓	✓	✓	✓	✓	✓	✓	✓	✓	✓	✓	✓		✓	✓	✓		✓	✓	✓	✓	✓	✓	✓	✓	✓	✓	✓	✓	✓	✓	✓	93.75%
Age of the animals	✓		✓		✓					✓		✓		✓	✓		✓										✓		✓				31.25%
Description of genetic modification status (Knock-out, transgenic, SPF)																																	0.0%
Information related to previous procedures performed on the animals														✓																			0.0%

Table S2. (Continued)

References	Bastos, et al., 2011	Shukla, et al., 1999	Muralidhar, et al., 2011	Süntar, et al., 2013	Mekonnen, et al. 2013	Pieters, et al., 1995	Korkina, et al., 2007	Bigoniya, et al., 2013	Lodhi, et al., 2011	Tabandeh, et al., 2013	Sonmez, et al., 2015	Karakaş, et al., 2012	Choi, et al., 2001	Parente, et al., 2011	Olugbuyiro, et al., 2010	Süntar, et al., 2010	Kim, et al., 2013	Chaudhari, et al., 2006	Swamy, et al., 2006	Hernandes, et al., 2010	Sidhu, et al., 1999	Paramesha, et al., 2015	Nagappan, et al., 2012	Qu, et al., 2013	Ghosh, et al., 2012	Mukherjee, et al., 2013	Melo, et al., 2011	Ahamed, et al., 2008	Zyuz'kov, et al., 2012	Singh, et al., 2005	Sharath, et al., 2010	Vidya, et al., 2012	
**Housing and husbandry**																																	
Housing of experimental animals (type of facility, type of cage or housing, material, number of cage companions)	✓	✓				✓	✓	✓	✓	✓		✓		✓	✓			✓		✓	✓		✓	✓	✓		✓	✓			✓		59.37%
Husbandry conditions (breeding programme, ligth/dark cycle, temperature, of water)				✓					✓	✓				✓		✓						✓	✓	✓									21.87%
Welfare-related assessements and interventions that were carried out before, during, or after the experiment																																	0.0%
**Sample size**																																	
Total number of animals used in each experimental and the number f animals in each experimental group	✓		✓		✓	✓	✓	✓	✓	✓	✓	✓	✓	✓	✓	✓	✓	✓	✓	✓		✓	✓	✓	✓		✓	✓		✓	✓	✓	84.37%
Explanation regarding the decision of the number of animals and details of sample size calculation						✓																											3.12%
**Allocation of animals into experimental groups**																																	
Full details of how animals were allocated to experimental groups (including randomisation or matching)	✓								✓					✓							✓		✓	✓								✓	21.87%
Order in which the animals in the different experimental groups were treated and assessed	✓		✓		✓	✓	✓	✓	✓	✓	✓		✓		✓	✓	✓	✓	✓	✓	✓	✓	✓	✓	✓			✓			✓	✓	75.0%
**Experimental outcomes**																																	
Clear experimental outcomes assessed	✓					✓	✓	✓	✓	✓		✓		✓		✓	✓	✓			✓	✓	✓		✓	✓	✓	✓	✓	✓			62.5%
**Statistical methods**																																	
Statistical methods used for each analysis	✓	✓	✓	✓	✓	✓	✓	✓	✓	✓	✓	✓	✓	✓	✓	✓	✓	✓	✓	✓	✓	✓	✓	✓	✓	✓	✓	✓	✓	✓	✓	✓	100.0%
Specification of the unit of analysis for each dataset		✓	✓	✓			✓			✓	✓	✓	✓	✓	✓	✓	✓	✓	✓		✓	✓		✓	✓	✓			✓		✓	✓	68.75%
Methods used to assess whether the data met the assumptions of the statistical approach																																	0.0%

Table S2. (Continued)

References	Bastos, et al., 2011	Shukla, et al., 1999	Muralidhar, et al., 2011	Süntar, et al., 2013	Mekonnen, et al. 2013	Pieters, et al., 1995	Korkina, et al., 2007	Bigoniya, et al., 2013	Lodhi, et al., 2011	Tabandeh, et al., 2013	Sonmez, et al., 2015	Karakaş, et al., 2012	Choi, et al., 2001	Parente, et al., 2011	Olugbuyiro, et al., 2010	Süntar, et al., 2010	Kim, et al., 2013	Chaudhari, et al., 2006	Swamy, et al., 2006	Hernandes, et al., 2010	Sidhu, et al., 1999	Paramesha, et al., 2015	Nagappan, et al., 2012	Qu, et al., 2013	Ghosh, et al., 2012	Mukherjee, et al., 2013	Melo, et al., 2011	Ahamed, et al., 2008	Zyuz’kov, et al., 2012	Singh, et al., 2005	Sharath, et al., 2010	Vidya, et al., 2012	
**Results**																																	
**Baseline data**																																	
Description of the animals health status of animals, for each experimental grup, before treatment																														□			0.0%
**Number analysed**																																	
Number or animals in each group included in each analysis (absolute numbers)	✓	□	✓	✓	✓	✓	✓	✓	✓	✓	✓	✓	✓	✓	✓	✓	✓	✓	✓	✓	□	✓	✓	✓	✓	□	□	✓	□	✓	✓	✓	84.37%
Animals or data not included in the analysis (and explanation for the exclusion)																																	0.0%
**Outcomes and estimation**																																	
Information (Mean= Standard Deviation)	□	✓	✓	✓		□	✓	□	□	✓	✓	✓	✓	✓	✓	✓	✓	✓	✓	□	✓	✓	□	✓	✓	✓	□		✓		✓	✓	68.75%
**Adverse events**																																	
Information on mortality of experimental animals (Mean=Standard Deviation)																																	0.0%
Modifications to the experimental protocols made to reduce adverse events																																	0.0%
**Discussion**																																	
**Interpretation /scientific implications**																						□											
Interpretatio of the results, taking into account the study objectives and hypotheses,current theory and relevant studies	□	✓	✓	✓	✓	✓	□	✓	✓	✓	□	□	□	✓	✓	□	✓	✓	✓	□	□	✓	✓	✓	✓	✓	✓	✓	✓	✓	✓	✓	75.0%
Comments on the study limitations (sources of bias, limitations of the animal model, imprecision associated with the results)				□																					□								0.0%
**Generalisability /translation**																																	
Comments on how the findings are likely to translate to other species or systems, including relevance to human biology				□																			□		□								0.0%
**Funding**																																	
List of funding sources and the role of the funder(s) in the studt	✓	✓	□	✓	✓	✓	□	□	✓	✓	✓	✓	□	✓	✓	✓	✓	✓	✓	✓	✓	✓	✓		✓	✓	✓	✓	□	✓	✓	✓	81.25%
**Results**	20	18	21	24	19	20	23	20	22	27	17	24	17	26	22	23	21	25	22	18	19	19	25	23	25	17	21	20	16	19	24	21	

✓ Means that the criteria have been met according to Arrive. The percentage was calculated considering the total number of studies that met the criteria included in the bias analysis.
